# Estimating the overdispersion in COVID-19 transmission using outbreak sizes outside China

**DOI:** 10.12688/wellcomeopenres.15842.3

**Published:** 2020-07-10

**Authors:** Akira Endo, Centre for the Mathematical Modelling of Infectious Diseases COVID-19 Working Group, Sam Abbott, Adam J. Kucharski, Sebastian Funk

**Affiliations:** 1 Department of Infectious Disease Epidemiology, London School of Hygiene & Tropical Medicine, London, WC1E 7HT, UK; 2 The Alan Turing Institute, London, NW1 2DB, UK; 3 Centre for the Mathematical Modelling of Infectious Diseases, London School of Hygiene & Tropical Medicine, London, WC1E 7HT, UK

**Keywords:** COVID-19, SARS-CoV-2, novel coronavirus, overdispersion, superspreading, branching process

## Abstract

**Background:** A novel coronavirus disease (COVID-19) outbreak has now spread to a number of countries worldwide. While sustained transmission chains of human-to-human transmission suggest high basic reproduction number *R*_0_, variation in the number of secondary transmissions (often characterised by so-called superspreading events) may be large as some countries have observed fewer local transmissions than others.

**Methods:** We quantified individual-level variation in COVID-19 transmission by applying a mathematical model to observed outbreak sizes in affected countries. We extracted the number of imported and local cases in the affected countries from the World Health Organization situation report and applied a branching process model where the number of secondary transmissions was assumed to follow a negative-binomial distribution.

**Results:** Our model suggested a high degree of individual-level variation in the transmission of COVID-19. Within the current consensus range of *R*_0_ (2-3), the overdispersion parameter *k* of a negative-binomial distribution was estimated to be around 0.1 (median estimate 0.1; 95% CrI: 0.05-0.2 for R0 = 2.5), suggesting that 80% of secondary transmissions may have been caused by a small fraction of infectious individuals (~10%). A joint estimation yielded likely ranges for *R*_0_ and *k* (95% CrIs: *R*_0_ 1.4-12; *k* 0.04-0.2); however, the upper bound of *R*_0_ was not well informed by the model and data, which did not notably differ from that of the prior distribution.

**Conclusions: **Our finding of a highly-overdispersed offspring distribution highlights a potential benefit to focusing intervention efforts on superspreading. As most infected individuals do not contribute to the expansion of an epidemic, the effective reproduction number could be drastically reduced by preventing relatively rare superspreading events.

## Introduction

A novel coronavirus disease (COVID-19) outbreak, which is considered to be associated with a market in Wuhan, China, is now affecting a number of countries worldwide ^1, 2^. A substantial number of human-to-human transmission has occurred; the basic reproduction number *R*_0_ (the average number of secondary transmissions caused by a single primary case in a fully susceptible population) has been estimated around 2–3 ^3– 5^. More than 100 countries have observed confirmed cases of COVID-19. A few countries have already been shifting from the containment phase to the mitigation phase ^6, 7^, with a substantial number of locally acquired cases (including those whose epidemiological link is untraceable). On the other hand, there are countries where a number of imported cases were ascertained but fewer secondary cases have been reported than might be expected with an estimated value of *R*_0_ of 2–3.

This suggests that not all symptomatic cases cause a secondary transmission, which was also estimated to be the case for past coronavirus outbreaks (SARS/MERS) ^8, 9^. High individual-level variation (i.e. overdispersion) in the distribution of the number of secondary transmissions, which can lead to so-called superspreading events, is crucial information for epidemic control ^9^. High variation in the distribution of secondary cases suggests that most cases do not contribute to the expansion of the epidemic, which means that containment efforts that can prevent superspreading events have a disproportionate effect on the reduction of transmission.

We estimated the level of overdispersion in COVID-19 transmission by using a mathematical model that is characterised by *R*_0_ and the overdispersion parameter *k* of a negative binomial branching process. We fit this model to worldwide data on COVID-19 cases to estimate *k* given the reported range of *R*_0_ and interpret this in the context of superspreading.

## Methods

### Data source

We extracted the number of imported/local cases in the affected countries ( Table 1) from the WHO situation report 38 ^10^ published on 27 February 2020, which was the latest report of the number of imported/local cases in each country (as of the situation report 39, WHO no longer reports the number of cases stratified by the site of infection). As in the WHO situation reports, we defined imported cases as those whose likely site of infection is outside the reporting country and local cases as those whose likely site of infection is inside the reporting country. Those whose site of infection was under investigation were excluded from the analysis (Estonia had no case with a known site of infection and was excluded). In Egypt and Iran, no imported cases have been confirmed, which cause the likelihood value to be zero; data in these two countries were excluded. To distinguish between countries with and without an ongoing outbreak, we extracted daily case counts from an online resource ^11^ and determined the dates of the latest case confirmation for each country (as of 27 February).

**Table 1.** The number of confirmed COVID-19 cases reported (as of 27 February 2020).

Country	Total cases	Imported cases	Local cases	Site of infection unknown	Deaths	Latest date of case confirmation
South Korea	1766	17	605	1144	13	27/02/2020
Japan	186	39	129	18	3	27/02/2020
Singapore	93	24	69	0	0	27/02/2020
Australia	23	20	3	0	0	26/02/2020
Malaysia	22	20	2	0	0	27/02/2020
Vietnam *	16	8	8	0	0	13/02/2020
Philippines *	3	3	0	0	1	05/02/2020
Cambodia *	1	1	0	0	0	30/01/2020
Thailand	40	23	7	10	0	26/02/2020
India *	3	3	0	0	0	03/02/2020
Nepal *	1	1	0	0	0	24/01/2020
Sri Lanka	1	1	0	0	0	27/01/2020
USA	59	56	2	1	0	26/02/2020
Canada	11	9	1	1	0	27/02/2020
Brazil	1	1	0	0	0	26/02/2020
Italy	400	3	121	276	12	27/02/2020
Germany	21	3	14	4	0	27/02/2020
France	18	8	7	3	2	27/02/2020
UK	13	12	1	0	0	27/02/2020
Spain	12	10	1	1	0	27/02/2020
Croatia	3	2	1	0	0	26/02/2020
Austria	2	2	0	0	0	27/02/2020
Finland	2	2	0	0	0	26/02/2020
Israel	2	2	0	0	0	27/02/2020
Russia *	2	2	0	0	0	31/01/2020
Sweden	2	2	0	0	0	27/02/2020
Belgium *	1	1	0	0	0	04/02/2020
Denmark	1	1	0	0	0	27/02/2020
Estonia ^†^	1	0	0	1	0	27/02/2020
Georgia	1	1	0	0	0	26/02/2020
Greece	1	1	0	0	0	27/02/2020
North Macedonia	1	1	0	0	0	26/02/2020
Norway	1	1	0	0	0	27/02/2020
Romania	1	1	0	0	0	26/02/2020
Switzerland	1	1	0	0	0	27/02/2020
Iran ^†^	141	0	28	113	22	27/02/2020
Kuwait	43	43	0	0	0	27/02/2020
Bahrain	33	33	0	0	0	26/02/2020
UAE	13	8	5	0	0	27/02/2020
Iraq	6	6	0	0	0	27/02/2020
Oman	4	4	0	0	0	27/02/2020
Lebanon	1	1	0	0	0	27/02/2020
Pakistan	2	1	0	1	0	26/02/2020
Afghanistan	1	1	0	0	0	24/02/2020
Egypt * ^†^	1	0	1	0	0	14/02/2020
Algeria	1	1	0	0	0	25/02/2020

^*^ Countries considered to be without an ongoing outbreak

† Countries excluded from analysis

### Model

Assuming that the offspring distributions (distribution of the number of secondary transmissions) for COVID-19 cases are identically- and independently-distributed negative-binomial distributions, we constructed the likelihood of observing the reported number of imported/local cases (outbreak size) of COVID-19 for each country. The probability mass function for the final cluster size resulting from *s* initial cases is, according to Blumberg *et al*. ^12^, given by

$$c(x;s)=P(X=x;s)=\frac{ks}{kx+x-s}\left(\begin{array}{c} kx+x-s\\ x-s \end{array}\right)\frac{{\left(\frac{R_{0}}{k}\right)}^{x-s}}{{\left(1+\frac{R_{0}}{k}\right)}^{kx+x-s}}.$$

If the observed case counts are part of an ongoing outbreak in a country, cluster sizes may grow in the future. To address this issue, we adjusted the likelihood for those countries with ongoing outbreak by only using the condition that the final cluster size of such a country has to be larger than the currently observed number of cases. The corresponding likelihood function is

$$c_{\text{o}}(x;s)=P(X\geq x;s)=1-\sum_{m=0}^{x-1}c(m;s),$$

with a convention $\sum_{m=0}^{-1}c(m;s)=0.$ We assumed that the growth of a cluster in a country had ceased if 7 days have passed since the latest reported case (denoted by set *A*). We applied the final size likelihood *c*( *x*; *s*) to those countries and *c*_o_( *x*; *s*) to the rest of the countries (countries with an ongoing outbreak: *B*). The total likelihood is

$$L(R_{0},k)=\prod_{i\in A}P(X=x_{i};s_{i})\prod_{i\in B}P(X\geq x_{i};s_{i}).$$

### Statistical analysis

Varying the assumed *R*_0_ between 0–5 (fixed at an evenly-spaced grid of values), we estimated the overdispersion parameter *k* using the likelihood function described above. We used the Markov-chain Monte Carlo (MCMC) method to provide 95% credible intervals (CrIs). The reciprocal of *k* was sampled where the prior distribution for the reciprocal was weakly-informed half-normal (HalfNormal( *σ* = 10)). We employed the adaptive hit-and-run Metropolis algorithm ^13^ and obtained 500 thinned samples from 10,000 MCMC steps (where the first half of the chain was discarded as burn-in). We confirmed that the final 500 samples have an effective sample size of at least 300, indicating sufficiently low auto-correlation.

We also performed a joint-estimation of *R*_0_ and *k* by the MCMC method (with a weakly-informed normal prior *N*( *μ* = 3, *σ* = 5) for *R*_0_ and the weakly-informed half-normal prior (HalfNormal( *σ* = 10)) for the reciprocal of *k*.

Statistical analysis was implemented in R-3.6.1 with a package { LaplacesDemon}-16.1.1. The reproducible code for this study is available on GitHub ^14^.

### Proportion responsible for 80% of secondary transmissions

Using the estimated *R*_0_ and *k*, we computed the estimated proportion of infected individuals responsible for 80% of the total secondary transmissions. Such proportion *p*_80%_ is given as

$$1-p_{80\%}=\int_{0}^{X}\text{NB}\left(\left\lfloor x\right\rfloor ;k,\frac{k}{R_{0}+k}\right)\;dx,$$

where *X* satisfies

$$1-0.8=\frac{1}{R_{0}}\int_{0}^{X}\left\lfloor x\right\rfloor \text{NB}\left(\left\lfloor x\right\rfloor ;k,\frac{k}{R_{0}+k}\right)\;dx.$$

Here, $\text{NB}\left(x;k,\frac{k}{R_{0}+k}\right)$ represents the probability mass of a negative-binomial distribution with a mean *R*_0_ and an overdispersion parameter *k*. This calculation is eased by the following rearrangement:

$$\frac{1}{R_{0}}\int_{0}^{X}\left\lfloor x\right\rfloor \text{NB}\left(\left\lfloor x\right\rfloor ;k,\frac{k}{R_{0}+k}\right)\;dx=\int_{0}^{X-1}\text{NB}\left(\left\lfloor x\right\rfloor ;k+1,\frac{k}{R_{0}+k}\right)\;dx.$$

We computed *p*_80%_ for each MCMC (Markov-chain Monte Carlo) sample to yield median and 95% CrIs.

### Model comparison with a Poisson branching process model

To test if our assumption of overdispersed offspring distribution better describes the data, we compared our negative-binomial branching process model with a Poisson branching process model, which assumes that the offspring distribution follows a Poisson distribution instead of negative-binomial. Since a negative-binomial distribution converges to a Poisson distribution as *k* → ∞, we approximately implemented a Poisson branching process model by fixing *k* of the negative-binomial model at 10 ^10^. We compared the two models by the widely-applicable Bayesian information criterion (WBIC) ^15^.

### Simulation of the effect of underreporting

We used simulations to investigate potential bias caused by underreporting, one of the major limitations of the present study. Underreporting in some countries may be more frequent than others because of limited surveillance and/or testing capacity, causing heterogeneity in the number of cases that could have affected the estimated overdispersion. See *Extended data* (Supplementary materials) ^16^ for detailed methods.

### The effect of a differential reproduction number for imported cases

Due to interventions targeting travellers (e.g. screening and quarantine), the risk of transmission from imported cases may be lower than that from local cases. As part of the sensitivity analysis in *Extended data*, we estimated *k* assuming that the reproduction number of imported cases is smaller than that of local cases.

## Results

**Figure 1.**
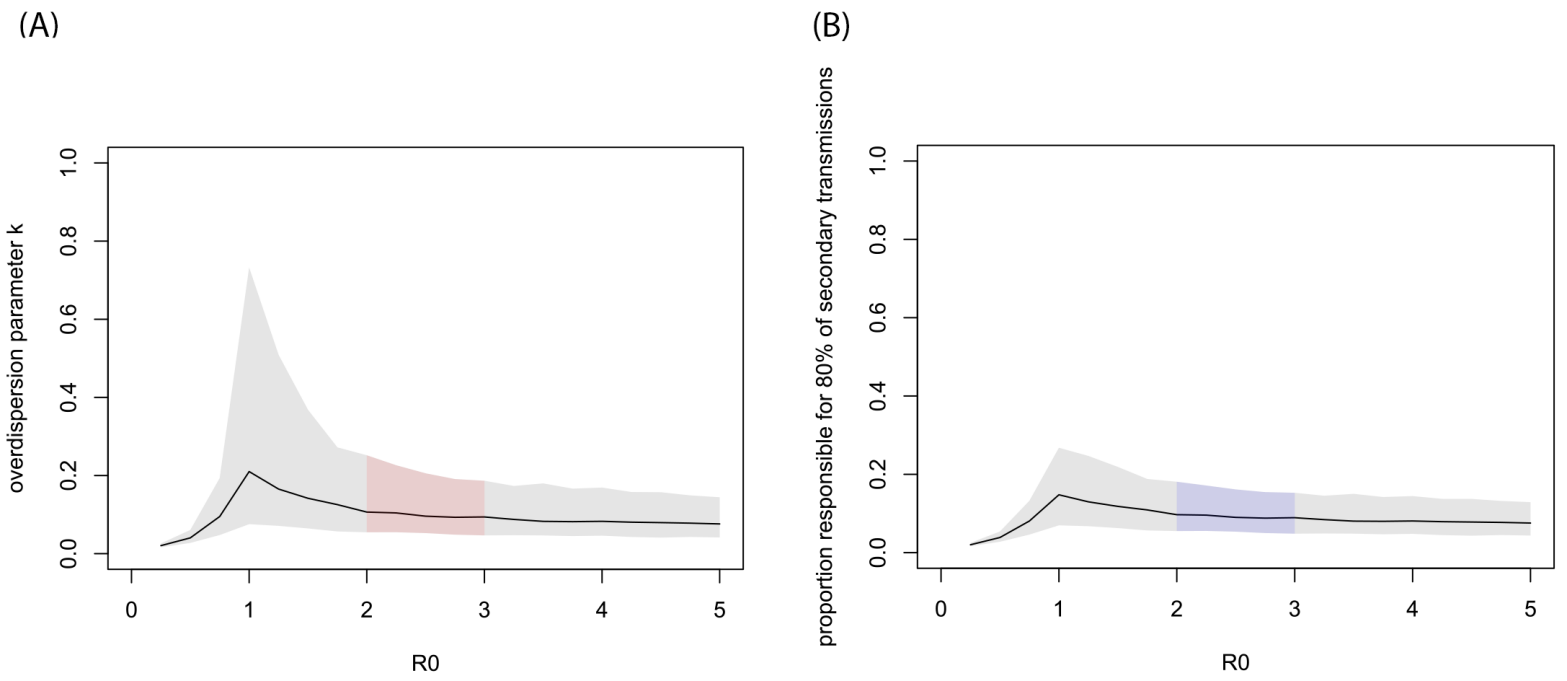
MCMC estimates given assumed *R*_0_ values. ( **A**) Estimated overdispersion parameter for various basic reproduction number *R*_0_. ( **B**) The proportion of infected individuals responsible for 80% of the total secondary transmissions ( *p*_80%_). The black lines show the median estimates given fixed *R*_0_ values and the grey shaded areas indicate 95% CrIs. The regions corresponding to the likely range of *R*_0_ (2–3) are indicated by colour.

**Figure 2.**
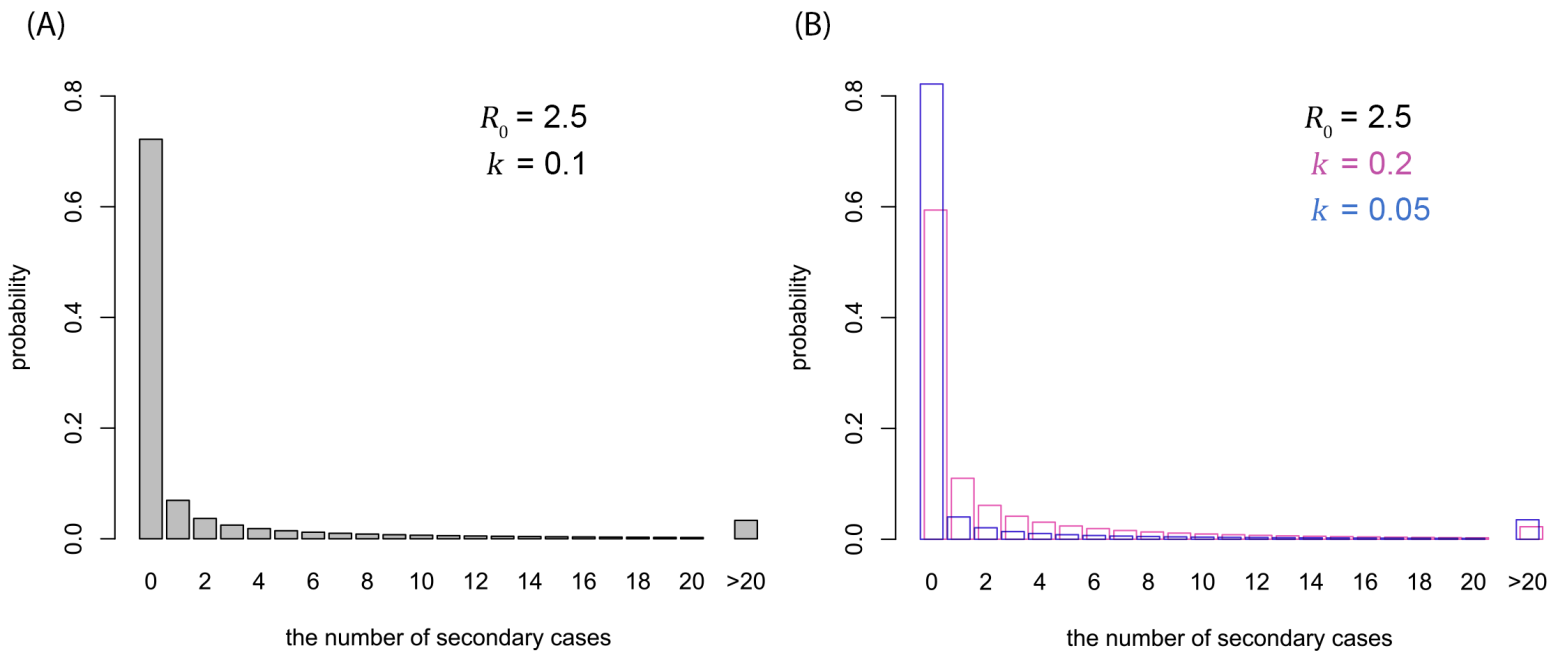
Possible offspring distributions of COVID-19. ( **A**) Offspring distribution corresponding to *R*_0_ = 2.5 and *k* = 0.1 (median estimate). ( **B**) Offspring distribution corresponding to *R*_0_ = 2.5 and *k* = 0.05 (95% CrI lower bound), 0.2 (upper bound). The probability mass functions of negative-binomial distributions are shown.

Our estimation suggested substantial overdispersion ( *k* ≪ 1) in the offspring distribution of COVID-19 ( Figure 1A and Figure 2). Within the current consensus range of *R*_0_ (2–3), *k* was estimated to be around 0.1 (median estimate 0.1; 95% CrI: 0.05–0.2 for *R*_0_ = 2.5). For the *R*_0_ values of 2–3, the estimates suggested that 80% of secondary transmissions may have been caused by a small fraction of infectious individuals (~10%; Figure 1B).

The result of the joint estimation suggested the likely bounds for *R*_0_ and *k* (95% CrIs: *R*_0_ 1.4–12; *k* 0.04–0.2). The upper bound of *R*_0_ did not notably differ from that of the prior distribution (=13.5), suggesting that our model and the data only informed the lower bound of *R*_0_. This was presumably because the contribution of *R*_0_ to the shape of a negative-binomial distribution is marginal when *k* is small ( *Extended data*, Figure S1) ^16^. A scatterplot ( *Extended data*, Figure S2) ^16^ exhibited a moderate correlation between *R*_0_ and *k* (correlation coefficient -0.4).

Model comparison between negative-binomial and Poisson branching process models suggested that a negative-binomial model better describes the observed data; WBIC strongly supported the negative-binomial model with a difference of 11.0 ( Table 2). The simulation of the effect of underreporting suggested that possible underreporting is unlikely to cause underestimation of overdispersion parameter *k* ( *Extended data*, Figure S3) ^16^. A slight increase in the estimate of *k* was observed when the reproduction number for imported cases was assumed to be lower due to interventions ( *Extended data*, Table S1).

**Table 2.** Model comparison between negative-binomial and Poisson branching process models.

Model	Parameter 95% CrIs		WBIC	ΔWBIC
	*R*_0_	*k*		
Negative-binomial	1.4–12	0.04-0.2	45.6	0
Poisson	0.95–1.2	10 ^10^ (fixed)	56.6	11.0

## Discussion

Our results suggested that the offspring distribution of COVID-19 is highly overdispersed. For the likely range of *R*_0_ of 2–3, the overdispersion parameter *k* was estimated to be around 0.1, suggesting that the majority of secondary transmission may be caused by a very small fraction of individuals (80% of transmissions caused by ~10% of the total cases). These results are consistent with a number of observed superspreading events observed in the current COVID-19 outbreak ^17^, and also in line with the estimates from the previous SARS/MERS outbreaks ^8^.

The overdispersion parameter for the current COVID-19 outbreak has also been estimated by stochastic simulation ^18^ and from contact tracing data in Shenzhen, China ^19^. The former study did not yield an interpretable estimate of *k* due to the limited data input. In the latter study, the estimates of *R*_e_ (the effective reproduction number) and *k* were 0.4 (95% confidence interval: 0.3–0.5) and 0.58 (0.35–1.18), respectively, which did not agree with our findings. However, these estimates were obtained from pairs of cases with a clear epidemiological link and therefore may have been biased (downward for *R*_0_ and upward for *k*) if superspreading events had been more likely to be missed during the contact tracing.

Although cluster size distributions based on a branching process model are useful in inference of the offspring distribution from limited data ^12, 20^, they are not directly applicable to an ongoing outbreak because the final cluster size may not yet have been observed. In our analysis, we adopted an alternative approach which accounts for possible future growth of clusters to minimise the risk of underestimation. As of 27 February 2020, the majority of the countries in the dataset had ongoing outbreaks (36 out of 43 countries analysed, accounting for 2,788 cases of the total 2,816). Even though we used the case counts in those countries only as the lower bounds of future final cluster sizes, which might have only partially informed of the underlying branching process, our model yielded estimates with moderate uncertainty levels (at least sufficient to suggest that *k* may be below 1). Together with the previous finding suggesting that the overdispersion parameter is unlikely to be biased downwards ^21^, we believe our analysis supports the possibility of highly-overdispersed transmission of COVID-19.

A number of limitations need to be noted in this study. We used the confirmed case counts reported to WHO and did not account for possible underreporting of cases. Heterogeneities between countries in surveillance and intervention capacities, which might also be contributing to the estimated overdispersion, were not considered (although we investigated such effects by simulations; see *Extended data*, Figure S3) ^16^. Reported cases whose site of infection classified as unknown, which should in principle be counted as either imported or local cases, were excluded from analysis. Some cases with a known site of infection could also have been misclassified (e.g., cases with travel history may have been infected locally). The distinction between countries with and without ongoing outbreak (7 days without any new confirmation of cases) was arbitrary. However, we believe that our conclusion is robust because the distinction does not change with different thresholds (4–14 days), within which the serial interval of SARS-CoV-2 is likely to fall ^22, 23^.

Our finding of a highly-overdispersed offspring distribution suggests that there is benefit to focusing intervention efforts on superspreading. As most infected individuals do not contribute to the expansion of transmission, the effective reproduction number could be drastically reduced by preventing relatively rare superspreading events. Identifying characteristics of settings that could lead to superspreading events will play a key role in designing effective control strategies.

## Data availability

### Source data

Zenodo: Extended data: Estimating the overdispersion in COVID-19 transmission using outbreak sizes outside China. https://doi.org/10.5281/zenodo.3740348 ^16^.

This project contains the following source data taken from references 10 and 11:

- bycountries_27Feb2020.csv. (Imported/local case counts by country from WHO situation report 38 ^10^.)
- dailycases_international_27Feb2020.csv. (Daily case counts by country from COVID2019.app ^11^.)

### Extended data

Zenodo: Extended data: Estimating the overdispersion in COVID-19 transmission using outbreak sizes outside China. https://doi.org/10.5281/zenodo.3911576 ^16^.

This project contains the following extended data

- supplementarymaterials.pdf. (Supplementary material: Estimating the amount of superspreading using outbreak sizes of COVID-19 outside China.)
- figS1.tif. (Figure S1. Offspring distributions for different *R*_0_ values. The probability mass functions of negativebinomial distributions are shown. The overdispersion parameter *k* is fixed at 0.1.)
- figS2.tif. (Supplementary Figure 2. Scatter plot of MCMC samples from a joint estimation of *R*_0_ and *k*. The dotted line represents the threshold *R*_0_ = 1)
- figS3.tif. (Supplementary Figure 3. Estimates of overdispersion from simulations with underreporting. (A) Maximum-likelihood estimates (MLEs) of overdispersion parameter k with different distributions for country-specific reporting probability *q _i_* (including constant *q _i_* = 1). Both imported and local cases are assumed to be reported at probability *q _i_* in country *i*. The blue dotted line indicates the true value *k* = 0.1. (B) MLEs where imported cases were assumed to be fully reported and local cases were reported at probability *q _i_*. (C) Probability density functions for beta distributions used in the simulation.)

### Code availability

**The reproducible code is available at:** https://github.com/akira-endo/COVID19_clustersize.

**Archived code at time of publication:** https://doi.org/10.5281/zenodo.3741743 ^14^.

**License:** MIT.

## References

[1] Zhu N Zhang D Wang W: A Novel Coronavirus from Patients with Pneumonia in China, 2019. *N Engl J Med.* 2020;382(8):727–733. 10.1056/NEJMoa2001017 31978945 PMC7092803

[2] Lai CC Shih TP Ko WC: Severe acute respiratory syndrome coronavirus 2 (SARS-CoV-2) and coronavirus disease-2019 (COVID-19): The epidemic and the challenges. *Int J Antimicrob Agents.* 2020;55(3): 105924. 10.1016/j.ijantimicag.2020.105924 32081636 PMC7127800

[3] Zhao S Lin Q Ran J: Preliminary estimation of the basic reproduction number of novel coronavirus (2019-nCoV) in China, from 2019 to 2020: A data-driven analysis in the early phase of the outbreak. *Int J Infect Dis.* 2020;92:214–217. 10.1016/j.ijid.2020.01.050 32007643 PMC7110798

[4] Zhang S Diao M Yu W: Estimation of the reproductive number of novel coronavirus (COVID-19) and the probable outbreak size on the Diamond Princess cruise ship: A data-driven analysis. *Int J Infect Dis.* 2020;93:201–204. 10.1016/j.ijid.2020.02.033 32097725 PMC7110591

[5] Abbott S Hellewell J Munday J: The transmissibility of novel Coronavirus in the early stages of the 2019-20 outbreak in Wuhan: Exploring initial point-source exposure sizes and durations using scenario analysis [version 1; peer review: 1 approved]. *Wellcome Open Res.* 2020;5:17. 10.12688/wellcomeopenres.15718.1 32322691 PMC7156988

[6] Headquarters for Novel Coronavirus Disease Control; Ministry of Health Labour and Welfare: Basic Policies for Novel Coronavirus Disease Control.2020 Reference Source

[7] Department of Health and Social Care, Hancock M: Press release: Government outlines further plans to support health and social care system in fight against COVID-19.2020; [cited 9 Mar 2020]. Reference Source

[8] Kucharski AJ Althaus CL: The role of superspreading in Middle East respiratory syndrome coronavirus (MERS-CoV) transmission. *Euro Surveill.* 2015;20(25):14–8. 10.2807/1560-7917.es2015.20.25.21167 26132768

[9] Lloyd-Smith JO Schreiber SJ Kopp PE: Superspreading and the effect of individual variation on disease emergence. *Nature.* 2005;438(7066):355–359. 10.1038/nature04153 16292310 PMC7094981

[10] World Health Organization: Coronavirus disease 2019 (COVID-19) Situation Report – 38.2020 Reference Source

[11] COVID2019.app - LIVE stats and graphs.2020; [cited 4 Mar 2020]. Reference Source

[12] Blumberg S Funk S Pulliam JR: Detecting differential transmissibilities that affect the size of self-limited outbreaks. Wilke CO, editor. *PLoS Pathog.* 2014;10(10):e1004452. 10.1371/journal.ppat.1004452 25356657 PMC4214794

[13] Chen MH Schmeiser B: Performance of the Gibbs, Hit-and-Run, and Metropolis Samplers. *J Comput Graph Stat.* 1993;2(3):251–272. 10.2307/1390645

[14] Endo A Abbott S Kucharski AJ: Estimating the amount of superspreading using outbreak sizes of COVID-19 outside China (Version v1.0.0). *Zenodo.* 2020 10.5281/zenodo.3741743

[15] Watanabe S: A Widely Applicable Bayesian Information Criterion.2013;14:867–897. 10.1088/0953-8984/23/18/184115

[16] Endo A Abbott S Kucharski AJ: Extended data: Estimating the overdispersion in COVID-19 transmission using outbreak sizes outside China. *Zenodo.* 2020 10.5281/zenodo.3911576 PMC7338915 32685698

[17] Liu Y Eggo RM Kucharski AJ: Secondary attack rate and superspreading events for SARS-CoV-2. *Lancet.* 2020;395(10227):e47. 10.1016/S0140-6736(20)30462-1 32113505 PMC7158947

[18] Riou J Althaus CL: Pattern of early human-to-human transmission of Wuhan 2019 novel coronavirus (2019-nCoV), December 2019 to January 2020. *Euro Surveill.* 2020;25(4): 2000058. 10.2807/1560-7917.ES.2020.25.4.2000058 32019669 PMC7001239

[19] Bi Q Wu Y Mei S: Epidemiology and Transmission of COVID-19 in Shenzhen China: Analysis of 391 cases and 1,286 of their close contacts. *medRxiv.* 2020; 2020.03.03.20028423. 10.1101/2020.03.03.20028423 PMC7185944 32353347

[20] Blumberg S Lloyd-Smith JO: Inference of R(0) and transmission heterogeneity from the size distribution of stuttering chains.Ferguson N, editor. *PLoS Comput Biol.* 2013;9(5):e1002993. 10.1371/journal.pcbi.1002993 23658504 PMC3642075

[21] Lloyd-Smith JO: Maximum Likelihood Estimation of the Negative Binomial Dispersion Parameter for Highly Overdispersed Data, with Applications to Infectious Diseases. Rees M, editor. *PLoS One.* 2007;2(2):e180. 10.1371/journal.pone.0000180 17299582 PMC1791715

[232] Li Q Guan X Wu P: Early Transmission Dynamics in Wuhan, China, of Novel Coronavirus-Infected Pneumonia. *N Engl J Med.* 2020;382(13):1199–1207. 10.1056/NEJMoa2001316 31995857 PMC7121484

[23] Nishiura H Linton NM Akhmetzhanov AR: Serial interval of novel coronavirus (COVID-19) infections. *Int J Infect Dis.* 2020;93:284–286. 10.1016/j.ijid.2020.02.060 32145466 PMC7128842

