# Supplementary material for: Estimating the overdispersion in COVID-19 transmission using outbreak sizes outside China
Source: Wellcome Open Res. 2020 Jul 10;5:67. Originally published 2020 Apr 9. [Version 3] doi: 10.12688/wellcomeopenres.15842.3 (PMC7338915; doi:10.12688/wellcomeopenres.15842.3)
Supplement: Peer review history

# Peer review: Reviewer response for version 3

**DOI:** 10.21956/wellcomeopenres.17714.r39515

**Published:** 2020-07-13

**Authors:** Lin Wang

**Affiliations:** 1 Mathematical Modelling of Infectious Diseases Unit, UMR2000, CNRS, Institut Pasteur, Paris, France

Nice revision.

Is the work clearly and accurately presented and does it cite the current literature?

Yes

If applicable, is the statistical analysis and its interpretation appropriate?

Yes

Are all the source data underlying the results available to ensure full reproducibility?

Yes

Is the study design appropriate and is the work technically sound?

Yes

Are the conclusions drawn adequately supported by the results?

Yes

Are sufficient details of methods and analysis provided to allow replication by others?

Yes

Reviewer Expertise:

Infectious disease modeling, epidemiology.

I confirm that I have read this submission and believe that I have an appropriate level of expertise to confirm that it is of an acceptable scientific standard.

# Peer review: Reviewer response for version 2

**DOI:** 10.21956/wellcomeopenres.17694.r39407

**Published:** 2020-07-07

**Authors:** Lin Wang

**Affiliations:** 1 Mathematical Modelling of Infectious Diseases Unit, UMR2000, CNRS, Institut Pasteur, Paris, France

The authors have addressed all comments raised in previous review.

Is the work clearly and accurately presented and does it cite the current literature?

Yes

If applicable, is the statistical analysis and its interpretation appropriate?

Yes

Are all the source data underlying the results available to ensure full reproducibility?

Yes

Is the study design appropriate and is the work technically sound?

Yes

Are the conclusions drawn adequately supported by the results?

Yes

Are sufficient details of methods and analysis provided to allow replication by others?

Yes

Reviewer Expertise:

Infectious disease modeling, epidemiology.

I confirm that I have read this submission and believe that I have an appropriate level of expertise to confirm that it is of an acceptable scientific standard.

# Peer review: Reviewer response for version 2

**DOI:** 10.21956/wellcomeopenres.17694.r39406

**Published:** 2020-07-06

**Authors:** Kaiyuan Sun

**Affiliations:** 1 Division of International Epidemiology and Population Studies, Fogarty International Center, National Institutes of Health, Bethesda, MD, USA

The authors have fully addressed the suggestions and I do not have further comments.

Is the work clearly and accurately presented and does it cite the current literature?

Yes

If applicable, is the statistical analysis and its interpretation appropriate?

Yes

Are all the source data underlying the results available to ensure full reproducibility?

Yes

Is the study design appropriate and is the work technically sound?

Partly

Are the conclusions drawn adequately supported by the results?

Partly

Are sufficient details of methods and analysis provided to allow replication by others?

Yes

Reviewer Expertise:

Infectious disease modeling.

I confirm that I have read this submission and believe that I have an appropriate level of expertise to confirm that it is of an acceptable scientific standard.

# Peer review: Reviewer response for version 1

**DOI:** 10.21956/wellcomeopenres.17377.r38601

**Published:** 2020-05-27

**Authors:** Kaiyuan Sun

**Affiliations:** 1 Division of International Epidemiology and Population Studies, Fogarty International Center, National Institutes of Health, Bethesda, MD, USA

In this study, the authors estimate the over-dispersion of SARS-CoV-2 transmission using imported and local COVID-19 cases reported during the early phase of the epidemic through fitting the outbreak size distribution of a over-dispersed branching process. The analysis is well executed. The manuscript is well written and the results are clearly presented. However, the data used in the study may have a few potential bias representing several alternative scenarios that I recommend the authors to explore:

1. The number of imported cases is likely an underestimate of the true number of cases as screening of travelers is unlikely to reach high detection rate for SARS-CoV-2 ^1^. Certain studies estimated the reporting rate is only around 30-40%, even for countries with high surveillance intensity ^2^. This is likely different from the reporting rate of local cases (see point 3). I recommend the authors explore reporting rate of imported cases and local cases separately.
2. The over-dispersion estimated for SARS in the previous study is under un-controlled epidemic scenario ^3^. However, for imported cases detected through travel screening, certain control measures is likely in-place such as isolation/quarantine, which will reduce the effective reproduction number, thus in Figure 1, the effective R0 range for imported cases could extend to <1.
3. It's also quite likely that local transmission is heavily under-reported during February as well. A way to gauge this under-detection is to see when each country reported the first few deaths due to COVID-19. Assuming an infection fatality of 1% will suggest a few hundred cumulative infections about 2 weeks before the detection of death. The authors already listed a number of deaths at the same date of case reporting, I recommend the authors also reports the number of deaths 2-weeks later (or the delay from case detection to death that the authors finds appropriate) and comment on the possible rate of under reporting for local cases, and together with point 1, how it may affects the estimates of k.

Is the work clearly and accurately presented and does it cite the current literature?

Yes

If applicable, is the statistical analysis and its interpretation appropriate?

Yes

Are all the source data underlying the results available to ensure full reproducibility?

Yes

Is the study design appropriate and is the work technically sound?

Partly

Are the conclusions drawn adequately supported by the results?

Partly

Are sufficient details of methods and analysis provided to allow replication by others?

Yes

Reviewer Expertise:

Infectious disease modeling.

I confirm that I have read this submission and believe that I have an appropriate level of expertise to confirm that it is of an acceptable scientific standard, however I have significant reservations, as outlined above.

## References

[1] : Estimated effectiveness of symptom and risk screening to prevent the spread of COVID-19. *eLife*.2020;9: 10.7554/eLife.55570 10.7554/eLife.55570 PMC706003832091395

[2] : Quantifying bias of COVID-19 prevalence and severity estimates in Wuhan, China that depend on reported cases in international travelers. *medRxiv*.2020; 10.1101/2020.02.13.20022707 10.1101/2020.02.13.20022707

[3] : Superspreading and the effect of individual variation on disease emergence. *Nature*.2005;438(7066) : 10.1038/nature04153 355-9 10.1038/nature04153 16292310PMC7094981

# Response

**Published:** 2020-06-30

**Authors:** Akira Endo

**Affiliations:** London School of Hygiene & Tropical Medicine, UK

Thank you for your constructive suggestions. We have discussed potential biases as suggested and performed additional analysis where applicable. Please find our responses below.

**1. The number of imported cases is likely an underestimate of the true number of cases as screening of travelers is unlikely to reach high detection rate for SARS-CoV-2. Certain studies estimated the reporting rate is only around 30-40%, even for countries with high surveillance intensity. This is likely different from the reporting rate of local cases (see point 3). I recommend the authors explore reporting rate of imported cases and local cases separately.**

> We agree that the number of imported cases is likely to be under ascertained. In our sensitivity analysis, we explored how the estimated value of k may be biased with different probabilities of reporting. We found that the same level of underreporting for both imported and local cases tend to result in overestimation of k, while the estimate was hardly affected if the imported cases are fully reported but the local cases are underreported. As the scenario suggested by the reviewer may likely to lie in between these two extreme scenarios we explored, we expect that such scenario would lead to the overestimation of k (but not as much as our “equally underreported” scenario; Figure S3A).

**2. The over-dispersion estimated for SARS in the previous study is under un-controlled epidemic scenario. However, for imported cases detected through travel screening, certain control measures is likely in-place such as isolation/quarantine, which will reduce the effective reproduction number, thus in Figure 1, the effective R0 range for imported cases could extend to <1.**

> We have included the suggested scenario as part of our sensitivity analysis. Assuming that R0 for local cases is 2.5, we varied the effective reproduction number for the imported cases (0.5, 0.8 and 1.2) and estimated the value of k. We found that the estimates of k were larger than those in our baseline analysis for R0 = 2.5 if the assumed reproduction number for the imported cases (R _I_) was below 1 (k = 0.3 for R _I_ = 0.5; k = 0.2 for R _I_ = 0.8). For R _I_ = 1.2, the estimate was similar to our baseline analysis (k = 0.1). We have added the description of this additional analysis in the supplementary document.

**3. It's also quite likely that local transmission is heavily under-reported during February as well. A way to gauge this under-detection is to see when each country reported the first few deaths due to COVID-19. Assuming an infection fatality of 1% will suggest a few hundred cumulative infections about 2 weeks before the detection of death. The authors already listed a number of deaths at the same date of case reporting, I recommend the authors also reports the number of deaths 2-weeks later (or the delay from case detection to death that the authors finds appropriate) and comment on the possible rate of under reporting for local cases, and together with point 1, how it may affects the estimates of k.**

> As the reviewer suggests, the number of deaths is a useful measure to assess underreporting. Assuming the average infection fatality 1% for the initial deaths may be subject to bias because the earliest cases may have specific age profiles that result in a different fatality. However, when averaged over the dataset, the overall case fatality may suggest the possible degree of underreporting in the dataset. As the mean lags of 8-13 days from case confirmation to death  have been used for early outbreaks in existing studies [1,2], we referred to the WHO situation report 45 (5 March) and 52 (12 March), published on the 7 ^th^ and 14 ^th^ day from the situation report 38 we used in the analysis [3,4]. The total number of deaths in the countries included in our analysis was 168 and 1,065, respecctively. Given 2,815 total confirmed cases as of February 27 ^th^, these suggest ascertainment ratios of 16.8% and 2.6%, respectively (assuming the true infection fatality risk is 1%). However, these ratios may be underestimates because of the rapid growth in both cases and deaths. It was suggested that the lag distribution from confirmation to death has a large variation (coefficient of variation 50%-100% [1,2]), and early-reported deaths from the cases confirmed later than February 27 ^th^ may have inflated the number of deaths in situation reports 45 and 52. In either case, we believe that the assumed reporting probability in our sensitivity analysis (Figure S3C) was consistent overall with these observations. Although we did not include the above calculation in the manuscript because it is only a rough estimation in which we are not completely confident, we cited Niehus et al. to show that our assumed range of the reporting probability in the sensitivity analysis was plausible:

(Supplementary document, Section 3) *An existing study suggested 38% as an optimistic global estimate of the detection probability for imported cases from Wuhan, China, with a substantial variation between countries [1].*

 

1. Mizumoto K, Chowell G. Estimating Risk for Death from Coronavirus Disease, China, January–February 2020. Emerg Infect Dis. 2020;26(6):1251-1256. https://dx.doi.org/10.3201/eid2606.200233
2. Russell TW, Hellewell J, Jarvis CI, et al. Estimating the infection and case fatality ratio for coronavirus disease (COVID-19) using age-adjusted data from the outbreak on the Diamond Princess cruise ship, February 2020. *Euro Surveill*. 2020;25(12):2000256. doi:10.2807/1560-7917.ES.2020.25.12.2000256
3.  World Health Organization: Coronavirus disease 2019 (COVID-19) Situation Report – 45. 2020. https://www.who.int/docs/default-source/coronaviruse/situation-reports/20200305-sitrep-45-covid-19.pdf
4. World Health Organization: Coronavirus disease 2019 (COVID-19) Situation Report – 52. 2020. https://www.who.int/docs/default-source/coronaviruse/situation-reports/20200312-sitrep-52-covid-19.pdf

# Peer review: Reviewer response for version 1

**DOI:** 10.21956/wellcomeopenres.17377.r38418

**Published:** 2020-04-28

**Authors:** Lin Wang

**Affiliations:** 1 Mathematical Modelling of Infectious Diseases Unit, UMR2000, CNRS, Institut Pasteur, Paris, France

In this manuscript, Endo *et al*. estimated the overdispersion of COVID-19 transmission outside of China. The authors collected the number of imported and local cases in each affected country from the World Health Organization situation report. Using likelihood-based inference, they fitted a negative-binomial or Poisson offspring distribution to the empirical data. In summary, this study is scientifically sound and well presented. I only have a few suggestions.

1. The authors may wish to add one or two sentences about the convergence of MCMC chains, such as the diagnosis used.
2. If I understood correctly, a thinning interval of 10 is used to sample the raw chains. With this thinning interval, is the auto-correlation sufficiently small?
3. As to the statistical model, it seems that the authors assumed that all imported cases arrived and triggered the local epidemic at the same time. If the cases arrived at different time points, will the inferred results be different? This manuscript might be useful to understand the effect of continuous seeding: Characterizing the dynamics underlying global spread of epidemics ^1^.

Is the work clearly and accurately presented and does it cite the current literature?

Yes

If applicable, is the statistical analysis and its interpretation appropriate?

Yes

Are all the source data underlying the results available to ensure full reproducibility?

Yes

Is the study design appropriate and is the work technically sound?

Yes

Are the conclusions drawn adequately supported by the results?

Yes

Are sufficient details of methods and analysis provided to allow replication by others?

Yes

Reviewer Expertise:

Infectious disease modeling, epidemiology.

I confirm that I have read this submission and believe that I have an appropriate level of expertise to confirm that it is of an acceptable scientific standard.

## References

[1] : Characterizing the dynamics underlying global spread of epidemics. *Nature Communications*.2018;9(1) : 10.1038/s41467-017-02344-z 10.1038/s41467-017-02344-z PMC576876529335536

# Response

**Published:** 2020-06-30

**Authors:** Akira Endo

**Affiliations:** London School of Hygiene & Tropical Medicine, UK

Thank you for your comments. We have added some technical details for better clarity as suggested. Please find our responses to each point below. 

**1. The authors may wish to add one or two sentences about the convergence of MCMC chains, such as the diagnosis used.**

**2. If I understood correctly, a thinning interval of 10 is used to sample the raw chains. With this thinning interval, is the auto-correlation sufficiently small?**

> We used the effective sample size to assess the convergence and (the weakness of) autocorrelation. For clarity, we have added a line in Statistical analysis section: “We confirmed that the final 500 samples have an effective sample size of at least 300, indicating sufficiently low auto-correlation.”

**3. As to the statistical model, it seems that the authors assumed that all imported cases arrived and triggered the local epidemic at the same time. If the cases arrived at different time points, will the inferred results be different? This manuscript might be useful to understand the effect of continuous seeding: Characterizing the dynamics underlying global spread of epidemics.**

> We agree with the reviewer that continuous seeding is an important issue in time-series epidemic analysis. However, our approach was not sensitive to the assumption that all the imported cases arrived at the same point in time. Because we only imposed the condition that the final cluster size has to be at least the size of the currently observed number of cases, the temporal distribution of the imported cases does not change the likelihood function we used.
